# HMGB1 deficiency reduces H_2_O_2_‐induced oxidative damage in human melanocytes via the Nrf2 pathway

**DOI:** 10.1111/jcmm.13895

**Published:** 2018-10-19

**Authors:** Kuanhou Mou, Wei Liu, Yi Miao, Fang Cao, Pan Li

**Affiliations:** ^1^ Department of Dermatology The First Affiliated Hospital of Xi'an Jiaotong University Xi'an Shaanxi China; ^2^ Center for Translational Medicine The First Affiliated Hospital of Xi'an Jiaotong University Xi'an Shaanxi China; ^3^ Key Laboratory for Tumor Precision Medicine of Shaanxi Province The First Affiliated Hospital of Xi'an Jiaotong University Xi'an China

**Keywords:** HMGB1, melanocyte, oxidative stress, vitiligo

## Abstract

Oxidative stress leads to melanocyte death and has been implicated in the pathogenesis of vitiligo. The nuclear factor, E2‐related factor 2 (Nrf2), is a critical transcription factor in protecting cells from oxidative damage. High‐mobility group box 1 (HMGB1) is a chromatin‐associated nuclear protein and an extracellular damage‐associated molecular pattern molecule. Extracellular HMGB1 released from activated immune cells, necrotic or injured cells, becomes a proinflammatory mediator through binding to cell‐surface receptors of responding cells. In this study, we investigated the role of HMGB1 from melanocytes in the response to oxidative stress and the mechanism involved. We showed that HMGB1 is expressed by primary normal human epidermal melanocytes (NHEMs). H_2_O_2_ treatment increased cytoplasmic translocation and extracellular release of HMGB1. HMGB1 knockdown by small interfering RNA (siRNA) led to decreased apoptosis of NHEMs. HMGB1 inhibition enhanced the expression of Nrf2 and its target genes. The expression of Nrf2 and its downstream antioxidant genes was downregulated after the supernatant of H_2_O_2_‐treated NHEMs was added to HMGB1‐deficient cells. HMGB1 knockdown by siRNA suppressed the expression of the autophagosome marker, LC3, and enhanced p62 expression. Coimmunoprecipitation with Keap1 showed a reduced Nrf2‐Keap1 interaction and an increased p62‐Keap1 interaction under oxidative stress. These data demonstrated that external stimuli (eg, oxidative stress) may trigger autocrine HMGB1 translocation and release by melanocytes, suppressing the expression of Nrf2 and downstream antioxidant genes to induce melanocyte apoptosis, and thereby participate in the pathological process of vitiligo.

## 1 INTRODUCTION

Skin, the body's largest organ, is strategically located at the interface with the external environment where it detects, integrates, and responds to a diverse range of stressors. It has already been established that the skin is an important peripheral neuro‐endocrine‐immune organ that is tightly networked to central regulatory systems.^1^

Melanogenesis is under complex regulatory control by multiple agents interacting via pathways activated by receptor‐dependent and ‐independent mechanisms, in hormonal, auto‐, para‐, or intracrine fashion. Tyrosinase are the key elements in the regulation of melanogenesis. The presence of a large‐binding site of the transcription factor family in the gene demonstrates the complexity and precise control of melanogenesis. Within the context of the skin as a stress organ, melanogenic activity serves as a unique molecular sensor and transducer of noxious signals and as regulator of local homoeostasis.^2^

Vitiligo is a common acquired skin depigmentation disorder characterized by the death of melanocytes.^3^ The aetiology of vitiligo and the causes of melanocyte death are not fully understood, yet. The oxidative stress hypothesis suggests an imbalanced redox state of the vitiliginous skin. This results in dramatic production of reactive oxygen species (ROS), such as H_2_O_2_. ROS oxidize cell components, leading to melanocyte destruction and creating depigmented macules.^4^ Increased levels of H_2_O_2_ were detected in the epidermis of vitiligo patients.^5^, ^6^

The nuclear factor, E2‐related factor 2 (Nrf2), is a regulator of cellular resistance to oxidants. The Nrf2‐kelch‐like ECH‐associated protein 1 (Keap1) system is one of the major cellular defence mechanisms against oxidative and electrophilic stresses.^7^, ^8^, ^9^, ^10^ Under quiescent conditions, the transcription factor, Nrf2, is constitutively degraded through the ubiquitin‐proteasome pathway. Its binding partner, Keap1, is an adaptor of the ubiquitin ligase complex that targets Nrf2. Under oxidative stress, Nrf2 is rapidly released from Keap1 and translocates into the nucleus, where it binds to ARE and induces the phase II antioxidant genes.^11^ These genes encode haem oxygenase‐1 (HO‐1), catalase, superoxide dismutase, glutathione‐S‐transferase, glutathione peroxidase, NADH quinone oxidoreductase 1 (NQO1), glutamate‐cysteine ligase catalytic subunit (GCLC), and glutamyl cysteine ligase modulator subunit (GCLM), which are important antioxidants in melanocytes.^12^ p62/SQSTM1 (hereafter referred to as p62) interacts with the Nrf2‐binding site of Keap1 and competitively inhibits the Keap1‐Nrf2 interaction, which is responsible for the expression of a battery of genes encoding antioxidant proteins and anti‐inflammatory enzymes.^13^, ^14^ Nrf2 is a critical transcription factor in protecting melanocytes from oxidative damage.^15^ Dysfunction of the Nrf2 signalling pathway may result in increased sensitivity of vitiligo melanocytes to H_2_O_2_‐induced oxidative insult.^6^

High‐mobility group box 1 (HMGB1) is a highly conserved nuclear protein involved in transcriptional activation and DNA folding in eukaryotic cells.^16^ Exposure to pathogens activates the highly conserved innate immune response, which triggers the release of HMGB1 from monocytes, macrophages, and other cells at the frontline of the host defence. HMGB1 translocates from the nucleus to the cytosol, where it accumulates in intracellular vesicles prior to secretion.^17^ Extracellular HMGB1 interacts with multiple receptors including the receptor for advanced glycation end products (RAGE),^18^ Toll‐like receptor 4 (TLR4) and TLR2. Oxidative stress such as that induced by H_2_O_2_ induces HMGB1 secretion from macrophages and monocytes.^19^ HMGB1 also regulates autophagy in response to oxidative stress. Tang et al^20^ have reported that ROS trigger HMGB1 translocation to the cytosol during starvation‐mediated autophagy in fibroblasts.

Recently, Kim et al^21^ have suggested a potential role for HMGB1 from keratinocytes in melanogenesis and demonstrated that secretion of HMGB1 from neighbouring keratinocytes influences melanocyte survival and the expression of melanogenesis‐related molecules. It is not clear whether the melanocytes themselves produce HMGB1. In this study, we investigated the role of HMGB1 from melanocytes in the response to oxidative stress and the mechanism involved. We demonstrated that H_2_O_2_‐induced oxidative stress triggered HMGB1 release from melanocytes. Oxidative stress triggered autocrine HMGB1 translocation and release from melanocytes, leading to suppression of the expression of Nrf2 and downstream antioxidant genes to induce melanocyte apoptosis, and thereby participate in the pathological process of vitiligo.

## 2 MATERIALS AND METHODS

### 2.1 Skin specimens and ethics statement

Normal skin specimens (for primary human melanocyte culture) were obtained from six healthy individuals who underwent circumcision. In this study, foreskin was from Asiatic‐Chinese yellow race donors. The study was performed in accordance with the Declaration of Helsinki (1964) and its later amendments, and the protocol was approved by the Clinic Research Ethics Board of the First Affiliated Hospital of Xi'an Jiaotong University. All participants gave written informed consent prior to inclusion in the study.

### 2.2 NHEM cultures

The epidermis was separated from the dermis after an overnight incubation of skin samples in a 0.25% Dispase II solution (Roche Diagnostics USA, Indianapolis, IN, USA) in PBS at 4°C. To separate cellular elements, epidermal sheets were incubated for 30 minutes at 37°C in a solution of 0.25% trypsin in PBS. The cell suspension was then filtered through a 70‐mm cell strainer and then centrifuged at 500 *g* for 5 minutes to harvest cells. Normal human epidermal melanocytes (NHEMs) were cultured in Medium254 (Cascade Biologics/Invitrogen, Portland, OR, USA) supplemented with human melanocyte growth supplement (Cascade Biologics/Invitrogen) in the presence of 5% CO_2_. It must be noted that melanocytes used for the experiments were lightly pigmented and dendritic morphology did not change significantly during relatively short periods of incubation including 6 and 12 hours after 0.5 mM H_2_O_2_ treatment (Figure S1).

### 2.3 Antibodies and reagents

In this study, we used rabbit anti‐human HMGB1, anti‐human p62, anti‐human Nrf2 (Abcam, Shanghai, China), anti‐human LC3, anti‐human Keap1 (Cell Signaling Technology, Danvers, MA, USA), and Alexa488‐conjugated goat anti‐rabbit IgG (H+L) (Invitrogen, Carlsbad, CA, USA) antibodies. Analytical pure grade H_2_O_2_ was purchased from TianJin Chemical Reagent Factory (Tianjin, China). Propidium iodide (PI) was purchased from Sigma‐Aldrich (St. Louis, MO, USA). Recombinant human HMGB1 (rHMGB1) was purchased from R&D Systems Inc. (Minneapolis, MN, USA).

### 2.4 Cell viability assay

Cell viability was assayed by the Cell Counting Kit‐8 (CCK‐8) (KeyGen Biotech, Nanjing, China) according to the manufacturer's protocol. Briefly, NHEMs were plated in 96‐well plates at a density of 2.5 × 10^4^ cells per well and cultured for 24 hours. Then, the culture medium was carefully removed and cells were treated with multiple concentrations of H_2_O_2_ in culture medium for 24 hours. Ten microlitres of CCK‐8 solution were added to each well. Triplicate blank control wells (without cells) and untreated control wells (cells without H_2_O_2_ treatment) were included. Colour change was measured at 450 nm using a microplate reader (PerkinElmer, Waltham, MA, USA).

### 2.5 Small interfering RNA gene silencing

The *HMGB1* small interfering RNA (siRNA) (5′‐GCAGAUGACAAGCAGCCUUTT‐3′) and control (scrambled) siRNA (5′‐UUCUCCGAACGUGUCACGUTT‐3′) oligonucleotides were synthesized by GenePharma (Shanghai, China). A single dose of 30 nmol of siRNA was administrated to the cells at 60% confluency by transfection with 250 μL of Lipofectamine RNAiMAX in Opti‐MEM medium (Thermo Fisher Scientific, Waltham, MA, USA) according to the manufacturer's instructions. Knockdown efficiencies were tested by immunoblotting 48 hours after the siRNA transfection.

### 2.6 Western blot analysis

For western blots, equal volumes of cell lysate extracts were separated by SDS‐PAGE and then transferred to nitrocellulose membranes. Membranes were blocked overnight at 4°C in blocking buffer (5% nonfat dried milk in PBS, 0.1% Tween‐20), followed by incubation with various primary antibodies. Horseradish peroxidase‐conjugated anti‐IgG antibody diluted at 1:5000 was used to label the membrane‐bound antibodies. β‐actin was used as a loading control. An enhanced chemiluminescence system (Pierce, Thermo Fisher Scientific) was used to visualize the membranes.

For analysis of H_2_O_2_‐induced HMGB1 release, primary human melanocytes were cultured in serum‐free medium. The culture supernatants were collected 24 hours after 0.5 mM H_2_O_2_ stimulation and concentrated with a filter (Centricon 10 kDa; Millipore, Billerica, MA, USA). Equal volumes of supernatants were used for western blotting analysis. Signals were detected using ChemiDoc™ ImageLab 4.1 software (Bio‐Rad, Shanghai, China).

### 2.7 Apoptosis assay by flow cytometry

NHEMs were collected and washed twice with PBS and suspended in 200 μL of binding buffer and 10 μL of Annexin‐V‐FITC for 20 minutes in the dark. Then, 300 μL of binding buffer and 5 μL of PI buffer were added to each sample. Apoptotic cells were counted by flow cytometry (BD FACSCanto™ II; BD Biosciences, San Jose, CA, USA) with BD Diva software.

### 2.8 Immunofluorescence staining

For immunofluorescence, NHEMs were placed on glass slides for 30 minutes, permeabilized in 0.2% Triton X‐100 buffer for 15 minutes, blocked with 3% BSA for 2 hours, and then incubated with a primary antibody (rabbit anti‐human HMGB1) and a secondary antibody (Alexa488‐conjugated goat anti‐rabbit IgG) solution supplemented with DAPI. Slides were viewed with a confocal laser scanning biological microscope (Leica TCS SP5 II, Wetzlar, Germany) with a 60× (NA = 1.40) oil objective. Images were acquired using Leica Las AF Lite software, which came with the microscope, and analysed using Photoshop CS5.

### 2.9 Quantitative real‐time PCR

Total RNA was isolated from melanocytes using a kit (TaKaRa MiniBEST Universal RNA Extraction Kit, Otsu, Japan) according to the manufacturer's instructions. Total RNA was reverse‐transcribed into first‐strand cDNA and real‐time (RT) PCR was performed with SYBR Premix Ex Taq II (TaKaRa). The primers used for RT‐PCR are listed in Table 1. Relative expression levels of cytokines were calculated using the 2^−ΔΔCt^ method and normalized to the value of *GAPDH*.

**Table 1:** Real‐time PCR primers

Gene	Sequence (5′–3′)
*Nrf2*	F: CTTGGCCTCAGTGATTCTGAAGTG R: CCTGAGATGGTGACAAGGGTTGTA
*HO‐1*	F: CAGGAGCTGCTGACCCATGA R: AGCAACTGTCGCCACCAGAA
*NQO‐1*	F: GGATTGGACCGAGCTGGAA R: AATTGCAGTGAAGATGAAGGCAAC
*GCLC*	F: GAAGTGGATGTGGACACCAGATG R: TTGTAGTCAGGATGGTTTGCGATAA
*GCLM*	F: GGAGTTCCCAAATCAACCCAGA R: TGCATGAGATACAGTGCATTCCAA
*GAPDH*	F: ATGACATCAAGAAGGTGGTG R: CATACCAGGAATGAGCTTG

### 2.10 Immunoprecipitation analysis

Cells were lysed at 4°C in ice‐cold lysis buffer (50 mM Tris‐HCl, pH 7.4, 150 mM NaCl, 1% NP‐40, 0.5% sodium deoxycholate, 0.1% SDS, protease inhibitor cocktail). Lysates were cleared by centrifugation at 12 000 *g* for 10 minutes. Before immunoprecipitation (IP), samples were incubated overnight at 4°C with gentle shaking with 5 μg/mL of the appropriate antibody or an irrelevant IgG in the presence of protein A or G agarose/sepharose beads (Thermo Fisher Scientific). Immune complexes were washed extensively with PBS, and proteins were eluted by boiling in 2× SDS sample buffer. Proteins were assayed by western blotting.

### 2.11 Statistical analysis

Student's *t* test was used to determine significance of between‐group differences. The dose‐response analyses were conducted by one‐way ANOVA with Dunnett's multiple comparison test. All values are expressed as means ± SD, and differences were considered statistically significant when *P* < 0.05. All statistical analysis were performed by the GraphPad Prism (GraphPad Software Inc, San Diego, CA, USA).

## 3 RESULTS

### 3.1 HMGB1 protein expression in NHEMs

**Figure 1:**
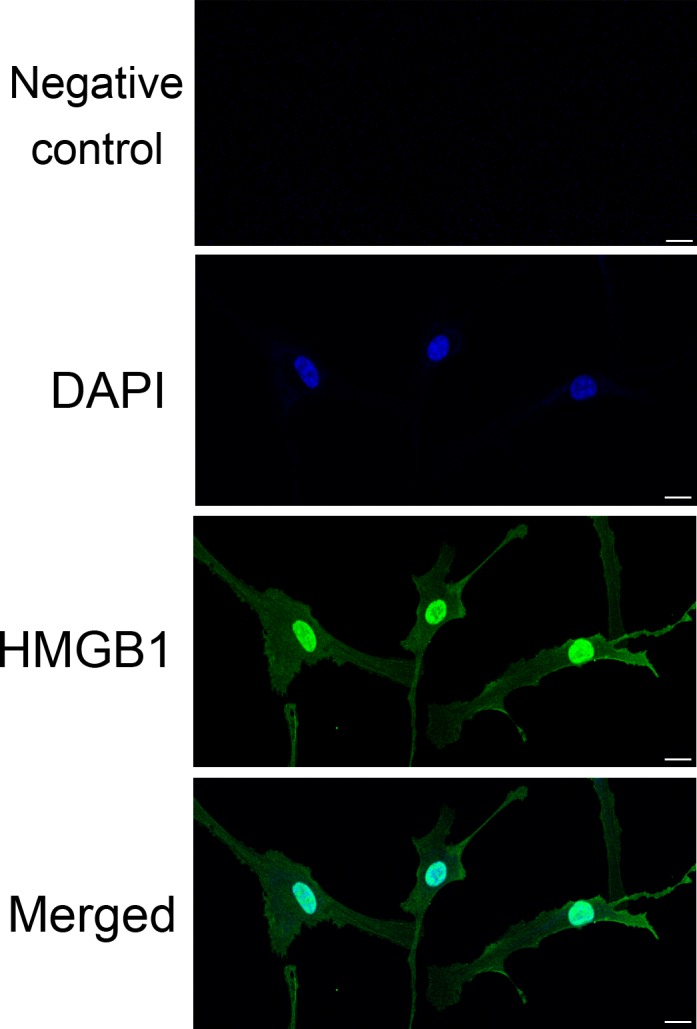
HMGB1 expression in NHEMs. Detection of HMGB1 by immunofluorescence using rabbit anti‐HMGB1 antibody followed by Alexa488‐conjugated secondary antibody (HMGB1, green; nucleus, blue). Scale bar = 10 μm. Three independent assays were performed

The expression of HMGB1 in NHEMs is shown in Figure 1. Immunofluorescence staining with anti‐HMGB1 showed that HMGB1 is predominantly located in the nucleus of NHEMs.

### 3.2 Cell viability after H_2_O_2_ treatment

**Figure 2:**
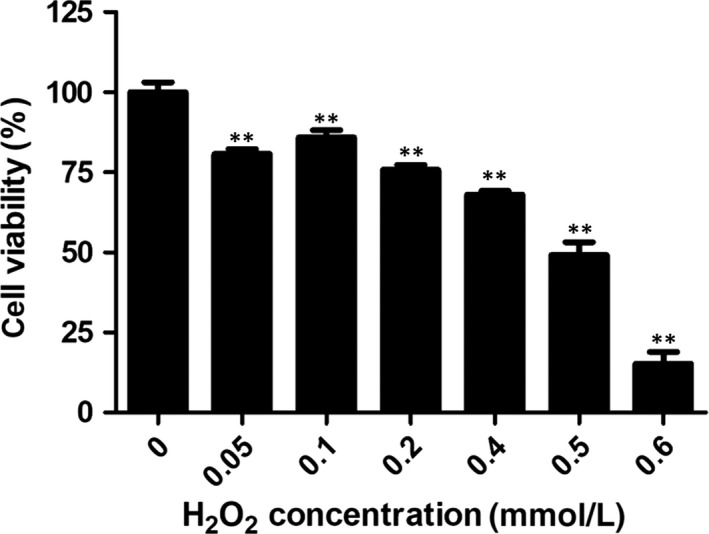
NHEM viability decreases after H_2_O_2_ treatment. Melanocytes were exposed to H_2_O_2_ at the indicated concentrations for 24 hours. A significant decline in cell viability was detected when the H_2_O_2_ concentration was ≥0.5 mM. Data are from three independent assays and are expressed as means ± SD. **P < 0.01

To investigate the viability of NHEMs in the presence of oxidative stress, we used H_2_O_2_ to induce oxidative stress. As shown in Figure 2, no remarkable decrease in cell viability was observed at low H_2_O_2_ doses (0.05, 0.1 mM), while higher doses (0.2, 0.4 mM) caused a statistically significant (*P* < 0.05) reduction in cell viability compared with untreated cells. The viability relative to untreated cells declined to 50.05 ± 7.35% when NHEMs were subjected to 0.5 mM H_2_O_2_ and the mean percentage of surviving cells fell to 15.23 ± 8.27% when the H_2_O_2_ dose was 0.6 mmol/L.

### 3.3 H_2_O_2_ treatment promotes cytoplasmic translocation and release of HMGB1 from NHEMs

**Figure 3:**
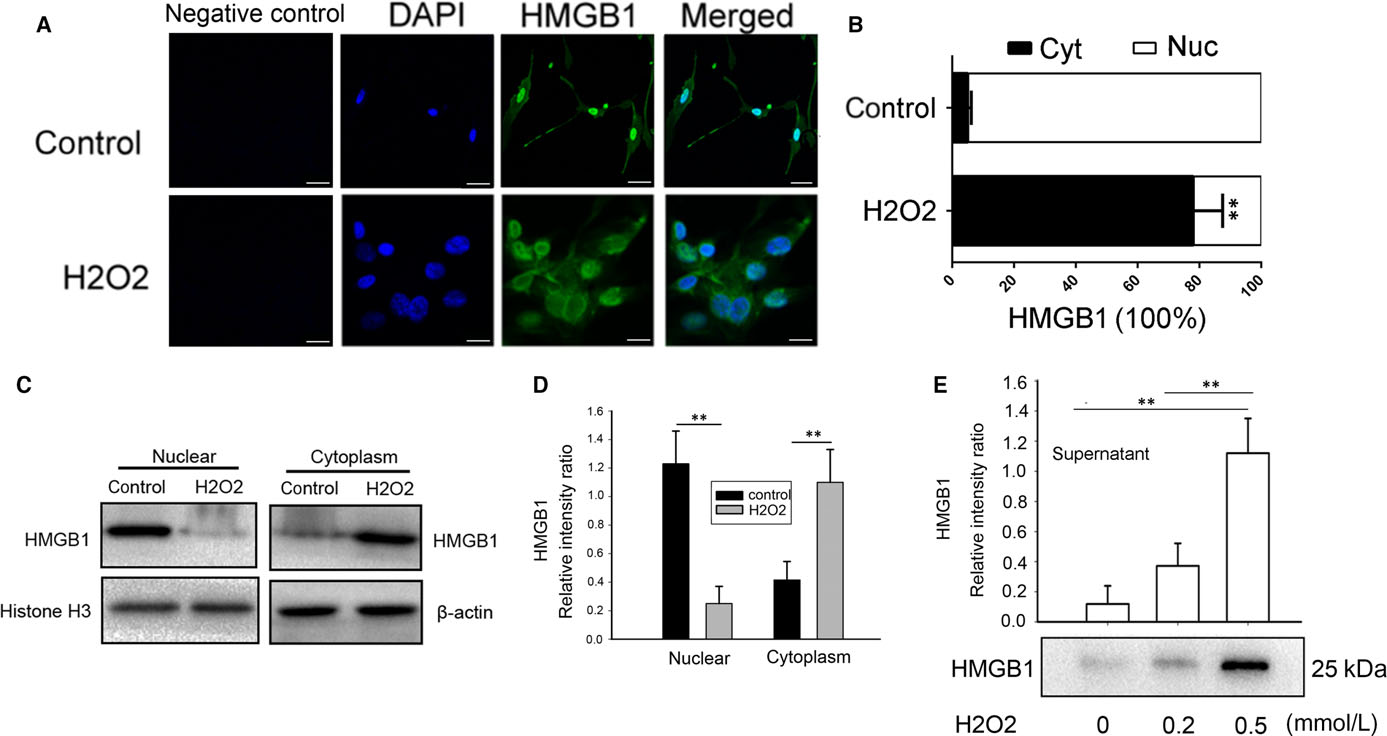
Effects of H_2_O_2_ on HMGB1 cytoplasmic translocation and release from NHEMs. (A) NHEMs were stimulated with 0.5 mM H_2_O_2_ for 12 hours and cells were immunostained with an HMGB1‐specific antibody (green) and DAPI (blue). (B) The mean nuclear (Nuc) and cytosolic (Cyt) HMGB1 staining intensity per cell was determined by cytometric imaging analysis as described in the Materials and Methods. (C, D) NHEM were stimulated with 0.5 mM H_2_O_2_ for 12 hours, HMGB1 content in the cytoplasmic and nuclear fraction was determined by western blotting analysis. Equal loading of samples was confirmed by western blotting analysis of each fraction with antibodies specific for a nuclear (Histone H3) or cytoplasmic (β‐actin) protein. (E) HMGB1 levels in the culture medium 12 hours after stimulation with H_2_O_2_ at the indicated doses. HMGB1 levels in the supernatant were determined by the relative optical intensity of the immunoreactive bands on western blots. Scale bar = 10 μm. Data are from three independent experiments. **P < 0.01

HMGB1 is an abundant nuclear protein with proinflammatory activity that depends on its extranuclear function. To investigate the distribution of HMGB1 under oxidative stress, immunofluorescence staining was used with specific anti‐HMGB1 antibodies. As shown in Figure 3A, untreated NHEMs primarily displayed nuclear localization of HMGB1. However, in cells treated with exogenous H_2_O_2_, there was an increase in the percentage of HMGB1 localization at the cytoplasm. To confirm HMGB1 cytoplasmic translocation, cytoplasmic and nuclear fractions of NHEMs were isolated and immunoblotted with antibodies specific for HMGB1 (Figure 3C). Consistently, levels of HMGB1 in the cytoplasmic fractions were increased dramatically after treatment with H_2_O_2_ (Figure 3D). Furthermore, the levels of HMGB1 released into the culture medium were measured by western blot analysis (Figure 3E). A low H_2_O_2_ dose (0.2 mM) slightly increased cellular HMGB1 protein levels; however, treatment with a high H_2_O_2_ dose (0.5 mM) dramatically increased the HMGB1 levels in the supernatant.

### 3.4 HMGB1 silencing inhibits H_2_O_2_‐induced NHEM apoptosis

**Figure 4:**
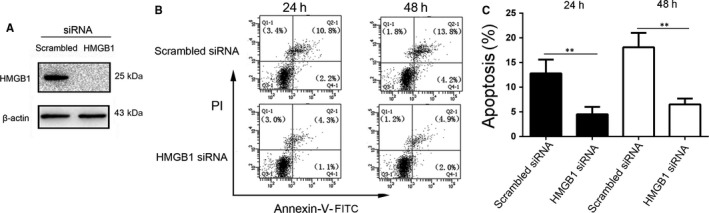
Depletion of HMGB1 inhibits H_2_O_2_‐induced apoptosis. (A) NHEMs were transfected with scrambled or HMGB1 siRNA, and knockdown efficiency was evaluated 48 hours later by western blotting. (B, C) Scrambled and HMGB1 siRNA‐transfected NHEMs were treated with 0.5 mM H_2_O_2_ and assayed for apoptosis 24 and 48 hours later by flow cytometry. **P < 0.01 vs scrambled group. The results shown are representative of three independent experiments with similar results. Data are expressed as means ± SD

HMGB1 release is a common mediator of the response to oxidative stress; consequently, we examined the role of HMGB1 in NHEMs under oxidative stress. We used siRNA targeting HMGB1, and HMGB1 knockdown efficiency was evaluated by western blotting (Figure 4A). In addition, using flow cytometry we examined apoptosis of cells transfected with scrambled siRNA or HMGB1 siRNA 24 and 48 hours after H_2_O_2_ treatment. As shown in Figure 4B, decreased apoptosis was observed in HMGB1 siRNA‐transfected cells compared with scrambled siRNA‐transfected cells. Collectively, these data suggest that HMGB1 plays a major role in promoting NHEM apoptosis.

### 3.5 HMGB1 knockdown enhances the expression of Nrf2 and its target genes, which can be reversed by addition of H_2_O_2_‐treated melanocyte supernatant or recombinant HMGB1 protein

**Figure 5:**
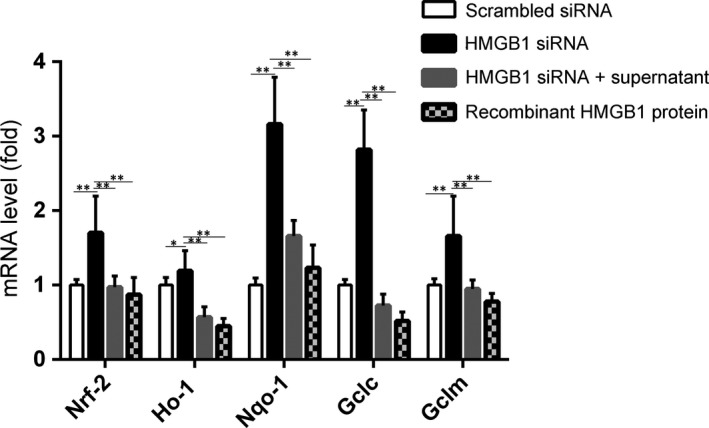
The effect of HMGB1 on *Nrf2* and its downstream genes. NHEMs were transfected with HMGB1 siRNA for 48 hours. The mRNA expression levels of *Nrf2* and its downstream antioxidant genes including *HO‐*1, *NQO‐1*,*GCLC*, and *GCLM* were detected by quantitative real‐time PCR. Addition of a supernatant ultrafiltrate from a melanocyte culture after treatment with 0.5 mM H_2_O_2_ for 24 hours or of recombinant human HMGB1 protein was followed by examination of the expression levels of *Nrf2* and its downstream antioxidant genes by real‐time PCR. Data are from three independent experiments. **P < 0.01

Nrf2‐ARE, a major antioxidant pathway, regulates oxidative stress‐related cytoprotective genes in melanocytes. We investigated whether knocking down HMGB1 affects the expression of Nrf2 and its downstream target genes. As show in Figure 5, quantitative RT‐PCR analysis showed that *Nrf2* and its target genes (*HO‐1*,* Nqo‐1*,* Gclc*,* Gclm*) were expressed at significantly higher levels in the HMGB1 siRNA group compared with the control. To confirm that HMGB1 can induce changes in *Nrf2* and its downstream genes, 48 hours after transfection of HMGB1 siRNA, the supernatant ultrafiltrate of 0.5 mM H_2_O_2_‐treated NHEMs was added for 24 hours. We found that compared with the HMGB1 transfection group, *Nrf2* and its downstream genes’ expression level was decreased. Similar results were obtained when recombinant HMGB1 protein was added.

### 3.6 HMGB1 silencing inhibits NHEM autophagy and enhances p62 expression

**Figure 6:**
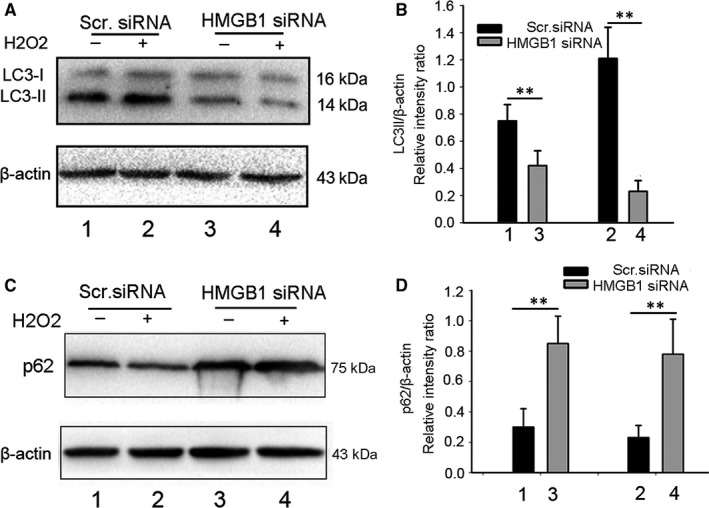
HMGB1 knockdown inhibits autophagy in NHEMs. (A–D) NHEMs transfected with scrambled or HMGB1 siRNA were treated with H_2_O_2_ for 12 hours, and LC3 and p62 were assayed by western blotting. **P < 0.01 vs scrambled group. Actin was used as a loading control. The data are from three independent experiments

It has been shown that HMGB1 translocation to the cytoplasm is closely associated with HMGB1 autophagy^20^ and that HMGB1 activated an autophagic response to oxidative stress.^22^ The selective autophagy substrate, p62, activates the stress‐responsive transcription factor, Nrf2, through inactivation of Keap1.^13^ To study the mechanism by which HMGB1 knockdown changes *Nrf2* and its downstream genes’ expression, we evaluated the expression of LC3 and p62, two characteristic markers of cell autophagy, in control and HMGB1‐knockdown cells. As shown in Figure 6A and B, H_2_O_2_ increased the expression levels of LC3II in NHEM control cells. However, the LC3II expression level in the HMGB1 siRNA group decreased significantly. In contrast, the p62 expression level in the HMGB1 siRNA group was significantly higher than that in the control group (Figure 6C and D).

### 3.7 HMGB1 knockdown suppresses Nrf2‐Keap1 interaction and induces p62‐Keap1 interaction under oxidative stress

To study the mechanism of the HMGB1 knockdown effect on the expression of Nrf2 and its downstream genes, we used co‐IP techniques to evaluate the potential Nrf2‐Keap1 and p62‐Keap1 interactions. The cells were treated with H_2_O_2_ after HMGB1 siRNA transfection. Co‐IP of Keap1 from NHEM lysates transfected with scrambled or HMGB1 siRNA showed that lower amounts of Nrf2 and higher amounts of p62 were coimmunoprecipitated with Keap1 in the latter compared with the former, indicating a decrease in Nrf2‐Keap1 and an increase in p62‐Keap1 interactions in response to oxidative stress. Thus, our data suggest that knockdown of HMGB1 results in increased p62 expression, which competes with Nrf2 on the interaction with Keap1, resulting in transcriptional activation of Nrf2 target genes.

## 4 DISCUSSION

HMGB1 is a DNA‐binding nonhistone protein that acts as a transcription regulator participating in DNA replication, recombination, transcription, and repair. In addition to its nuclear functions, extracellular HMGB1 released from activated immune cells, necrotic or injured cells, becomes a proinflammatory mediator through binding to cell‐surface receptors of responding cells. Recent studies have shown associations between HMGB1 and autoimmune diseases. However, its role in vitiligo has not been clarified, yet.

Herein, we demonstrated that HMGB1 secretion from NHEMs under oxidative stress (0.5 mM H_2_O_2_ treatment) affected melanocyte survival and the expression of *Nrf2* and its downstream genes. We propose that external stressors, such as oxidative stress, regarded as precipitating factors in vitiligo, stimulate HMGB1 release from NHEMs, leading to melanocyte apoptosis. This hypothesis was confirmed by siRNA gene silencing. Furthermore, HMGB1 knockdown enhanced the expression of *Nrf2* and its target genes, and the addition of exogenous H_2_O_2_‐treated melanocyte supernatant or recombinant HMGB1 protein reversed this effect. HMGB1 knockdown resulted in suppressed NHEM autophagy and in overexpression of p62, which competes with Nrf2 on the interaction with Keap1, resulting in transcriptional activation of Nrf2 target genes.

It has been reported that H_2_O_2_ stimulated macrophages and monocytes to actively release HMGB1,^23^ and that nuclear Hsp72 was a negative regulator of oxidative stress‐induced HMGB1 cytoplasmic translocation and release.^24^ These data showed that oxidative stress may be a common mechanism for regulating the translocation, release and activity of HMGB1, including in NHEMs. A couple of reports^21^, ^25^ have demonstrated that HMGB1 is expressed in keratinocytes and released from them under ultraviolet radiation or H_2_O_2_ treatment. As shown in Figures 1 and 3, HMGB1 is mainly expressed in the nucleus of NHEMs and is translocated from the nucleus to the cytoplasm after exposure to low doses of H_2_O_2_. Furthermore, HMGB1 was released into the culture medium after exposure to high doses of H_2_O_2_. The study of the autocrine effect of HMGB1 on melanocytes is of great significance to our understanding of the biological functions of melanocytes under oxidative stress.

As shown in Figure 4, HMGB1 silencing inhibited the H_2_O_2_‐induced NHEM apoptosis. It has been reported that HMGB1 plays a key role in apoptosis. Moreover, it has been shown to inhibit the death of yeast cells induced by Bak, a proapoptotic member of the Bcl‐2 family.^26^ Knocking down HMGB1 affected the expression of *Nrf2* and its downstream target genes, and addition of H_2_O_2_‐treated melanocyte supernatant or recombinant HMGB1 protein reversed this effect. This confirmed the role of autocrine HMGB1 in the expression of *Nrf2* and its downstream genes. Kim et al^21^ have reported that recombinant HMGB1 protein induced by external stimuli led to melanocyte apoptosis. The HMGB1 used in their experiments was exogenous recombinant HMGB1, which does not reflect the role of HMGB1 in melanocytes. Our results showed that upon oxidative stress, the alarmin, HMGB1, was produced by melanocytes, to induce apoptosis. This also explains the role of HMGB1 in the development of vitiligo and helps understand the mechanism of melanocyte death.

**Figure 7:**
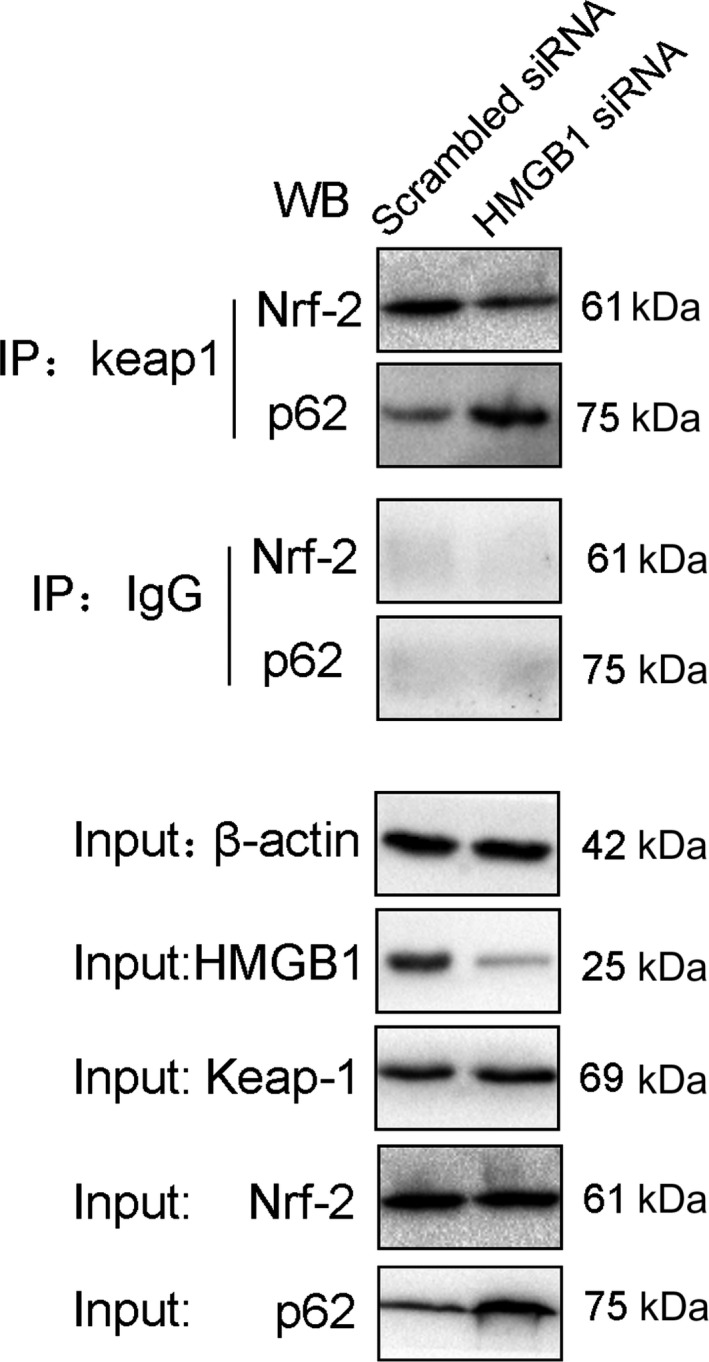
Nrf2 and p62 co‐IP with Keap1 after HMGB1 siRNA transfection. (A) Co‐IP of Nrf2 and p62 from NHEM lysates. NHEMs transfected with HMGB1 siRNA or scrambled siRNA were stimulated with H_2_O_2_ (0.5 mM) for 12 hours, and whole‐cell lysates were immunoprecipitated with antibodies specific for Keap1. IgG was used as a control. The precipitated complexes were separately immunoblotted with Nrf2 or p62‐specific antibodies. IP, immunoprecipitation; WB, western blotting. The blots are representative of three experiments with similar results

We further studied the mechanism of HMGB1 affecting the expression of *Nrf2* and its downstream genes. Our results suggest that HMGB1 can be transferred from the nucleus to the cytoplasm under oxidative stress (Figure 3). The literature indicates that HMGB1 can activate autophagy under oxidative stress. HMGB1 plays a location‐dependent role in inducing autophagy.^20^ Consistently, our results showed that after HMGB1 knockdown, autophagy was suppressed and p62 expression was increased. The competitive interaction of p62 with Keap1 can promote Nrf2 activation to play its antioxidant role. It has been reported that *ATG7* knockout in mice leads to p62 accumulation, and then to enhanced expression of Nrf2 target genes in melanocytes. As shown in Figure 7, in HMGB1‐deficient NHEMs, SQSTM1/p62 competed with Nrf2 for binding to its cytoplasmic anchor Keap1, leading to enhanced Nrf2 nuclear localization and transcription of its target genes. These results explain the relationship between HMGB1 and Nrf2 through autophagy.

It was reported that locally produced protective molecules such as melatonin also act on NRF2 in protection of melanocytes.^27^ Melatonin and its metabolites protect melanocytes from UVB‐induced DNA damage and oxidative stress through activation of NRF2‐dependent pathways; these actions are independent of an effect on the classic membrane melatonin receptors.^28^ Melatonin and its derivatives counteract the ultraviolet B radiation‐induced damage in human and porcine skin ex vivo.^29^ We believe that the study of the relationship between HMGB1 and melatonin is also significant for melanocytes under oxidative stress.

In conclusion, our data showed that oxidative stress may trigger autocrine HMGB1 translocation and release by melanocytes, reducing the expression of *Nrf2* and its downstream antioxidant genes to induce melanocyte apoptosis. We think that this is the mechanism by which HMGB1 participates in the pathological process of vitiligo.

## CONFLICT OF INTEREST

The authors confirm that they have no conflicts of interest.

## Supporting information

Figure 1

Figure 2

Data 1
