# Supplementary figures and images for: HMGB1 deficiency reduces H2O2‐induced oxidative damage in human melanocytes via the Nrf2 pathway
Source: J Cell Mol Med. 2018 Oct 19;22(12):6148–56. doi: 10.1111/jcmm.13895 (PMC6237592; doi:10.1111/jcmm.13895)

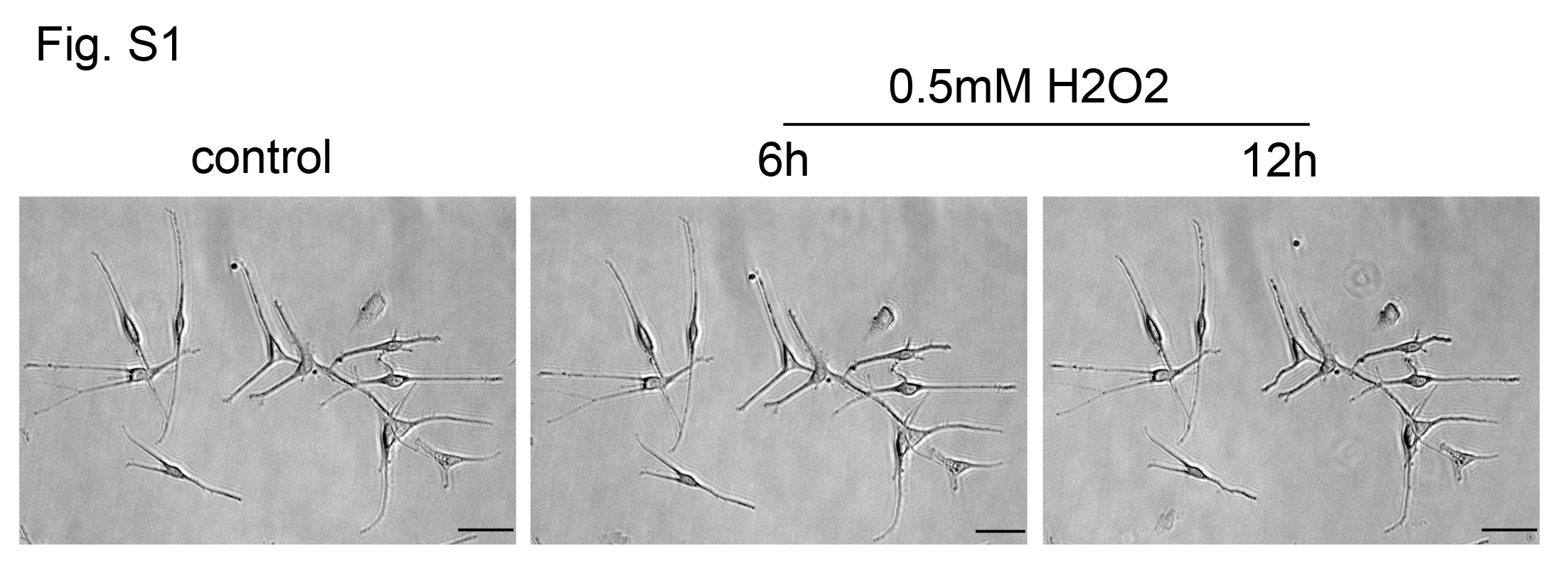

Supplement: Supplementary file 1 — Figure 1 [file JCMM-22-6148-s001.tif]

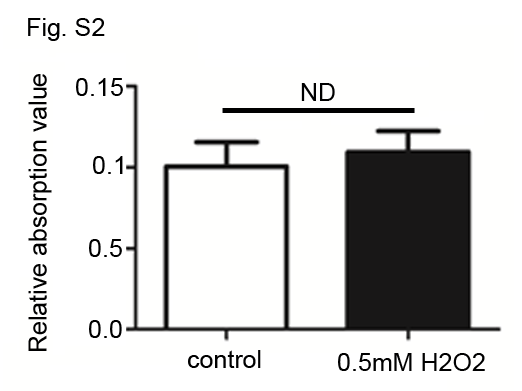

Supplement: Supplementary file 2 — Figure 2 [file JCMM-22-6148-s002.tif]
