# Supplementary material for: HMGB1 deficiency reduces H2O2‐induced oxidative damage in human melanocytes via the Nrf2 pathway
Source: J Cell Mol Med. 2018 Oct 19;22(12):6148–56. doi: 10.1111/jcmm.13895 (PMC6237592; doi:10.1111/jcmm.13895)
Supplement: Supplementary file 3 — Data 1 [file JCMM-22-6148-s003.doc]

Supplementary data

**Material and Methods:**

**Melanin content Assay**
NHEMs (5×10^5^ cells ) were plated for 24 hours and then treated with 0.5mM H_2_O_2_ for 12 hours. Cells were centrifuged and H_2_O_2_ was discarded and then cultured for 72 hours. The cells were centrifuged and pellets were solubilized in 100 μl of 1M NaOH in 80℃ for 2 hours to dissolve melanin, and the absorbance was measured spectrophotometrically at 405 nm by using a microplate reader (Perkins-Elmer, USA).

**Figure Legends:**

**Fig S1: Effects of H_2_O_2_ on morphology of normal human melanocytes**

Normal human melanocyte treated with 0.5mM H_2_O_2_ and cell morphology changes was observed at 6h, 12h after H_2_O_2_ treatment. Cell morphology did not change significantly when cells were exposed to H_2_O_2_. Representative pictures of cells were taken under the light microscope magnification 20x. Scale bar = 10 μm.

**FigS2: Effects of H_2_O_2_ on melanin pigmentation of normal human melanocytes**

NHEMs (5×10^6^ cells ) were plated for 24 hours and then treated with 0.5mM H_2_O_2_ for 12 hours. The cells were centrifuged and pellets were solubilized in 100 μl of 1M NaOH in 80℃ for 2 hours to dissolve melanin. A spectrophotometer with optical density set at 405 nm was used to detect optic absorptance. The data show the mean±SD of three separate experiments. ND: no difference
